# Voice deviation, dysphonia risk screening and quality of life in individuals with various laryngeal diagnoses

**DOI:** 10.6061/clinics/2018/e174

**Published:** 2018-03-07

**Authors:** Katia Nemr, Ariane Cota, Domingos Tsuji, Marcia Simões-Zenari

**Affiliations:** I Departamento de Fisioterapia, Fonoaudiologia e Terapia Ocupacional, Faculdade de Medicina (FMUSP), Universidade de Sao Paulo, Sao Paulo, SP, BR; II Departamento de Oftalmologia e Otorrinolaringologia, Faculdade de Medicina (FMUSP), Universidade de Sao Paulo, Sao Paulo, SP, BR

**Keywords:** Voice, Voice Disorders, Speech-Language Pathology, Medical History Taking, Quality of Life

## Abstract

**OBJECTIVES:**

To characterize the voice quality of individuals with dysphonia and to investigate possible correlations between the degree of voice deviation (D) and scores on the Dysphonia Risk Screening Protocol-General (DRSP), the Voice-Related Quality of Life (V-RQOL) measure and the Voice Handicap Index, short version (VHI-10).

**METHODS:**

The sample included 200 individuals with dysphonia. Following laryngoscopy, the participants completed the DRSP, the V-RQOL measure, and the VHI-10; subsequently, voice samples were recorded for auditory-perceptual and acoustic analyses. The correlation between the score for each questionnaire and the overall degree of vocal deviation was analyzed, as was the correlation among the scores for the three questionnaires.

**RESULTS:**

Most of the participants (62%) were female, and the mean age of the sample was 49 years. The most common laryngeal diagnosis was organic dysphonia (79.5%). The mean D was 59.54, and the predominance of roughness had a mean of 54.74. All the participants exhibited at least one abnormal acoustic aspect. The mean questionnaire scores were DRSP, 44.7; V-RQOL, 57.1; and VHI-10, 16. An inverse correlation was found between the V-RQOL score and D; however, a positive correlation was found between both the VHI-10 and DRSP scores and D.

**CONCLUSION:**

A predominance of adult women, organic dysphonia, moderate voice deviation, high dysphonia risk, and low to moderate quality of life impact characterized our sample. There were correlations between the scores of each of the three questionnaires and the degree of voice deviation. It should be noted that the DRSP monitored the degree of dysphonia severity, which reinforces its applicability for patients with different laryngeal diagnoses.

## INTRODUCTION

The voice is a multidimensional and fundamental phenomenon for oral communication. Voice assessment involves the compilation of such data as the history of the present complaint, signs and symptoms, use of the voice and related aspects 1,2. In addition to laryngoscopy, voice assessment includes auditory perceptual analysis (APA) and acoustic analysis (AA) by a speech therapist 2,3.

The Dysphonia Risk Screening Protocol (DRSP) is a clinical interview protocol that identifies voice disorders in individuals of all ages and genders 1. The factors associated with dysphonia have also been validated in the DRSP, indicating that this tool is a robust instrument for the screening, prevention, orientation, and assessment of voice disorders and can aid in treatment planning. This instrument provides relevant quantitative and qualitative information to health care professionals because its cutoff points indicate high and low dysphonia risk. The instrument was developed to be applied to individuals of any age and gender.

Speech therapy protocols that measure the impact of dysphonia on quality of life have recently gained attention in the international literature 4-12. When applied both before and after speech therapy, these protocols help to assess the effectiveness of interventions by measuring changes in patients' quality of life compared with their initial complaints. In this regard, the Voice-Related Quality of Life (V-RQOL) 4 measure and the Voice Handicap Index 10 (VHI-10) 5 are noteworthy. Both have been validated in the Brazilian Portuguese language 6,7. These scales are used globally in the clinical practice of speech therapy 8-12. A study of 37 patients with spasmodic dysphonia found a high correlation between the V-RQOL, VHI and VHI-10 and confirmed their usefulness for analyzing the effect of botulinum toxin application in this population 13. The authors note that these findings indicate that the choice of instrument may be based purely on the preference of the researcher/clinician 13.

Self-assessment instruments such as the VHI and V-RQOL are useful in voice therapy as they help the therapist assess the impact of the voice disorder on the patient's daily communication and seek strategies to minimize this impact 14.

In APA, the various aspects of voice production are assessed by a trained examiner. Characteristics such as roughness, breathiness, strain, asthenia, loudness, pitch, resonance, and other features are quantitatively and qualitatively assessed using a scale or protocol. The Consensus Auditory-Perceptual Evaluation of Voice (CAPE-V) 15,16 protocol is among the available instruments that stand out in the international literature. The latter analyzes the overall degree of voice deviation (D), roughness, breathiness, strain, pitch, loudness, and resonance and allows the examiner to describe other aspects, such as instability and asthenia. On a 100-mm line, the examiner is asked to mark the point that he/she considers to represent the degree of deviation for each aspect. This distance is then measured with a millimeter ruler to provide a numeric value that is recorded in a box to the right of the line.

In AA, various quantitative and qualitative data are collected using computer software 17. AA assumes that vocal acoustic signals validate auditory-perceptual signals and vice versa because AA allows the physiological and auditory-perceptual spheres to be integrated 18. Several vocal and acoustic components are correlated. For example, in the presence of laryngeal disorders, abnormal jitter is correlated with fundamental frequency measurements; furthermore, there is a correlation between abnormal shimmer measurements and disorders that interfere with voice intensity and between roughness and mass lesions in the vocal folds 19. However, AA may not be reliable in cases of severe vocal abnormalities given the limitations of currently available software; in addition, many acoustic measurements have no diagnostic value when they are considered separately 17.

The comprehensive application of the DRSP allows its use for dysphonia regardless of etiology and establishes an association between scores that indicate a high risk of dysphonia and voice disorder severity.

In addition, an investigation of the possible association between high-risk scores and the impact of dysphonia on quality of life is relevant because of the potential applications to clinical practice, teaching, and research as they pertain to therapeutic control and follow-up. An initial study that applied this instrument to children, adults and elderly subjects of both genders indicated high specificity and sensitivity for identifying the risk of dysphonia 1. In addition, it allowed an analysis of the relationship between drug use, overall degree of vocal deviation and the vocal symptoms of dry throat and shortness of breath, regardless of smoking habits/contact with smokers, the presence of comorbidities or hydration habits 20.

Children, adults and the elderly are affected by voice changes, but few studies have analyzed the causes and other epidemiological data in a population with a broad age range 21. A study of 2,019 male and female patients with dysphonia aged 1 to 18 years, 19 to 60 years and over 60 years found a higher prevalence of dysphonia among adult women. Additionally, the causes differed among age groups; vocal fold nodules and cysts predominated among children, functional dysphonia and gastroesophageal reflux among adults, and presbyphonia among the elderly 21.

Thus, there is interest in studying the relationship between the DRSP questionnaire and other well-established questionnaires that assess the impact of dysphonia on quality of life in patients with different voice disorders and a broad age range; additionally, there is interest in identifying the relationship between these questionnaires and the degree of voice deviation in this patient population.

The aims of the present study were to characterize the voice quality of individuals with dysphonia and to investigate possible correlations between the degree of voice deviation and the mean scores on the DRSP, the V-RQOL measure and the VHI-10.

## METHODS

This retrospective cross-sectional study was approved by the home institution's ethics committee (CAPPesq HCFMUSP 170/15).

The participants included 200 patients of all ages and genders who had dysphonia caused by various laryngeal and speech diagnoses determined at the Hospital das Clínicas da Faculdade de Medicina da Universidade de São Paulo (HCFMUSP), Ambulatory of Otorhinolaryngology. All the patients underwent laryngeal assessment, completed the study questionnaires, and underwent voice sample recording; afterward, they were sequentially and randomly included in our sample.

The voice recordings were conducted in an acoustically insulated room utilizing a desktop computer (Hewlett-Packard Company, United States) equipped with Audacity® software (Audacity Team, United States), an Edirol UA-101 interface (Roland, United Kingdom), and a unidirectional condenser headset microphone (AKG 520, Germany). The microphone was placed 3-5 cm from the subject's mouth at a 45- to 90-degree angle.

The following data were obtained for all patients in the study: age; laryngeal diagnosis; scores on the DRSP, V-RQOL measure, and VHI-10; and recorded voice samples based on CAPE-V tasks 16.

The APA in this study followed the CAPE-V protocol 15,16 and was administered by a speech therapist who specialized in voice disorders, had more than ten years of experience with this type of assessment, and had high internal reliability, with an intraclass correlation coefficient of 0.975 1.

The AA in this study was performed using the Praat software package developed by P. Boersma and D. Weenink from the Department of Phonetics at the University of Amsterdam (www.praat.org). The study measurements included the jitter/period perturbation quotient (PPQ, %), the shimmer/extent perturbation quotient (EPQ, %), and the harmonics-to-noise ratio (HNR, dB); these values were automatically extracted from the second emission of the vowel /a/, medial portion.

Spectrographic analyses were performed using Spectrogram software (Visualization Software, LLC). Abnormalities were defined as the presence of at least one of the following aspects: instability, subharmonic/frequency bifurcation, noise at high and/or low frequencies, breaks in frequency, voice breaks, and the absence of harmonics above 3.0 kHz.

The DRSP, V-RQOL, and VHI-10 were applied while the participants were waiting to have their voices recorded; subsequently, their scores were calculated and tabulated.

The overall DRSP score is calculated by totaling the scores of its 18 sub-items; this overall score ranges from zero to 131, with higher scores indicating a higher risk of dysphonia. One of the sub-items consists of a voice self-assessment in which the respondent is asked to mark the point that corresponds to their perceived amount of voice abnormality at the time of assessment on a 10-cm visual analogue scale (VAS) where zero (0) indicates no abnormality and ten (10) indicates maximal abnormality; afterward, this value is measured with a millimeter ruler. The following cutoff points specified an increased risk of dysphonia: 22.50 for children of both genders, 29.25 for adult women, 22.75 for adult men, and 27.10 for older adults of both genders 1. Based on the minimum and maximum values, the DRSP scores were divided into tertiles to compare the overall degree of vocal deviation (D on CAPE-V) in our subjects.

The V-RQOL comprises ten questions that investigate the physical and socio-emotional dimensions of voice disorders. Responses on this measure range from one (“not a problem”) to five (“as bad as it can be”); the overall score ranges from zero (maximum impact on quality of life) to 100 (no impact) 4,6.

The VHI-10 includes the ten most clinically relevant questions from the original Voice Handicap Index protocol 22. Responses on this index range from zero (never) to four (always); the overall score ranges from zero to 40, with higher scores indicating a greater voice handicap 7.

The laryngeal diagnoses, which were defined by the same team of otorhinolaryngologists, were later classified according to the type of dysphonia (behavioral or organic). Behavioral dysphonia (BD) is considered to be derived from the inappropriate use of the voice, including poor vocal technique, muscle tension, and vocal abuse/misuse 2, while organic dysphonia (OD) is the result of injuries to the muscles or nerves that regulate voice production and has no behavioral component 2. This categorization considered the history of the problem, and the presence of an organic lesion did not exclude patients from the BD group if the lesion was a clear consequence of vocal behaviors 2. As in the study by Behlau et al. 2, the BD group included patients with vocal fold edema, functional dysphonia, vestibular phonation, minor structural alterations, glottic gap, benign mass lesions, or normal examination in the presence of voice deviations; patients with OD presented vocal fold paralysis, laryngeal dystonia, postsurgical vocal fold scar, chronic laryngitis and/or laryngeal stenosis.

Statistical analyses included descriptive measures and the application of the following statistical tests: Spearman's correlation analysis, which was used to analyze the correlation between each questionnaire (DRSP, V-RQOL, and VHI-10) and D and any correlation among the three scales, and the Kruskal-Wallis test with Tukey's multiple comparison test, which was used to compare the DRSP score tertiles and D. The significance level was set at 5%.

## RESULTS

Of the 200 participants, 124 (62%) were female and 76 (38%) were male. The age of our participants ranged from 7 to 84 years; the mean age was 49 years (standard deviation [SD]=16.6 years) with the following distribution: 7 to 19 years – 16 patients (8%); 20 to 45 years – 58 patients (29%), 46 to 59 years – 69 patients (34%) and 60 years and older – 58 patients (29%).

All diagnoses were classified as either organic (79.5%) or behavioral (20.5%) dysphonia.

The APA results indicated that our patients had moderate dysphonia; their mean overall degree of voice deviation (D) was 59.54 (SD=18.10), and they had a mean predominance of roughness of 54.74 (SD=17.26). The means for the remainder of the APA parameters were as follows: breathiness, 44.57 (SD=19.46); strain, 25.39 (SD=25.89); loudness, 19.61 (SD=24.42) and that fifty-seven percent of the participants did not exhibit loudness abnormalities; and pitch, 26.07 (SD=25.41), where the majority (52%) of the pitch abnormalities were low-pitched vocalizations. Additionally, the following vocal aspects were also noted: asthenia (mean=1.17, SD=7.45), instability (mean=4.58, SD=16.45), bitonal voice (mean=1.12, SD=7.79), and hypernasal voice (mean=0.38, SD=5.30) (Table 1).

All the participants (100%) had at least one abnormal component on the AA, particularly the presence of noise at low frequencies (96.5%), abnormalities in the harmonious series (96.0%), the presence of noise at high frequencies (71.5%), a frequency to where the harmonics appeared well defined, a mean=1,426.7 Hz, and an average jitter of 1% (Tables 1 and 2).

The mean scores on the scales in our study were as follows: DRSP, 44.7 (SD=19.1); V-RQOL, 57.1 (SD=28.4); and VHI-10, 16.1 (SD=11.6). There was an inverse correlation between V-RQOL scores and D and a positive correlation between both VHI-10 and DRSP scores and D (Table 3).

Regarding the DRSP score tertiles, the first tertile was associated with a lower D, and the third tertile was associated with a higher D (Table 4).

There was a moderate correlation among the three scales in our study. The V-RQOL was inversely correlated with the VHI-10 and DRSP, indicating that higher voice-related quality of life was associated with lower voice handicap scores and a lower risk of dysphonia. A positive correlation was found between the VHI-10 and DRSP, indicating that higher voice handicap scores were associated with a higher risk of dysphonia (Table 5).

## DISCUSSION

The design of the present study allowed a thorough analysis and investigation of the applicability of the DRSP 1 in patients with dysphonia due to various causes. Voice disorders affect men and women, children, adults and the elderly 21. For this reason, our sample included individuals of both genders and a broad age range (7 to 84 years); however, most of the participants were female and exhibited organic dysphonia. According to reports in the scientific literature, in addition to the anatomical and physiological issues that predispose women to voice disorders 21, women seek medical care more often than men 23,24; furthermore, some voice disorders are more prevalent in female patients 25. Kopf et al. 17 analyzed 30 cases of dysphonia and found that the most common diagnoses in women were muscle tension, functional disorders, and mass lesions of the vocal folds. Other studies also found a greater prevalence of dysphonia in adult women 21,23.

Regarding age, the causes of dysphonia differ for each range; vocal fold nodules and cysts affect children more frequently, functional dysphonia and gastroesophageal reflux are most common in adults, and presbyphonia is most common among the elderly 21. The risk of dysphonia also varies for each age range 1. All age groups were represented in this study, but adults were most prevalent. This is explained by the fact that they face the greatest vocal demands, particularly during work, which can lead to a greater occurrence of voice-related complaints and concerns 23.

The results of our APA concur with previous studies; we observed a wide variability in the degree of voice deviation that was not related to the etiology of dysphonia 1,2,17.

The mean overall degree of voice deviation found in this study, which corresponds to moderate dysphonia, and the prevalence of roughness and breathiness were expected because the sample had various voice disorders 23 (Table 1). It is known that many individuals become motivated to seek care when they notice a more evident change in their voice 23.

All the participants exhibited at least one abnormal acoustic aspect, reinforcing the notion that AA integrates both the physiological and auditory-perceptual spheres in its assessment of voice 20. The presence of roughness and breathiness found on the APA is related to the high- and low-frequency noise observed on AA, and increased jitter values are expected in the presence of vocal fold lesions 19 (Tables 1 and 2).

The scores obtained in the questionnaires were outside the parameters of normality 1,4,5. These data indicate that the risk of dysphonia in these patients was much higher than the cutoff points specified for all age ranges and genders; furthermore, dysphonia had a moderate impact on the patients' quality of life.

In our study, the mean scores on the DRSP indicated an increased risk of dysphonia for all age and gender groups when the suggested cutoff points for this instrument were used 1. This finding reinforces the effectiveness of the DRSP to detect the dysphonia risk in laryngeal disorders that are characterized by patient complaints, acoustic anomalies, and auditory-perceptual abnormalities. In addition to the overall score, sub-item scores on the DRSP help clinicians identify the risk factors that contribute to the patient's condition and select the best treatment plan and patient guidelines 1,20. A study performed with the same instrument found a relationship between the drug use subscores and the occurrence of negative voice signs and symptoms 20.

The correlation between V-RQOL and D (Table 3) clearly provides evidence of the impact of dysphonia on patient quality of life 25,26,27, even when these scores were not especially high. The impact of dysphonia on different populations should also be considered. For example, patients who undergo total laryngectomy generally report a satisfactory quality of life; conversely, individuals whose profession relies on the use of their voice may report being greatly impacted by minor voice disorders. The present study did not aim to analyze the impact of dysphonia as a function of patients' profession; nevertheless, profession, gender, and age would be interesting to investigate in future studies 25.

As expected, the participants who had higher V-RQOL scores had lower scores on the VHI-10 (Table 5); this finding concurs with previous studies that applied the full and short versions of the Voice Handicap Index 13,28,29. The correlation between the V-RQOL and VHI-10 scales was moderate (Table 5), which concurs with a recent study 30. Together, these results reinforce the fact that these tools measure the impact of voice problems on patient quality of life in different ways, despite being similar instruments. In patients with presbyphonia and musculoskeletal stress syndrome, the V-RQOL 29 was found to be more sensitive than the VHI-10; however, the VHI-10 was more precise among patients with cysts 30. In the present study, the VHI-10 exhibited a greater correlation with the DRSP and D compared with the V-RQOL (Table 3).

The correlation between the DRSP and the other two scales and D (Table 3) reinforces its clinical applicability. Our analysis by tertiles showed a gradual increase in dysphonia that was parallel to higher scores on the DRSP (Table 4); in other words, higher degrees of voice deviation indicated a higher risk of dysphonia, thus confirming the robustness of this instrument. This is one of the strengths of the present study, as our initial hypothesis was confirmed. Moreover, in addition to providing a quantitative analysis via its scores, the DRSP also provides qualitative data that is relevant to better understanding the therapeutic process 1,20. Additionally, the value of the DRSP as a screening instrument and its indication in screenings and epidemiological studies was reinforced by relationship that was found between the DRSP scores and the degree of voice deviation. There is a need for this type of instrument in the epidemiological surveillance of voice and for studies that include a broad range of participants 31.

For individuals with voice complaints, voice self-perception may be more elucidative 14; this parameter is assessed in the DRSP. Additionally, the DRSP examines other factors related to dysphonia, such as smoking, negative voice-related signs and symptoms, hydration, and previous treatments 1,20,32.

The joint analysis of data from several instruments provides a thorough understanding of all the nuances of dysphonia, resulting in a more accurate assessment and treatment plan.

Many of our participants exhibited concomitant laryngeal diagnoses, such as Reinke's edema and cysts. As a continuation of this study, the classification of laryngeal diagnoses considering the presence of comorbidities and the respective vocal characteristics can elucidate the possible impacts and risks of each type of dysphonia.

The present study found a predominance of adult women, organic dysphonia, and moderate-grade voice deviation. The sample showed a high risk of dysphonia with a low-to-moderate impact on patient quality of life.

There was an inverse correlation between V-RQOL scores and the overall degree of voice deviation and a positive correlation between both the VHI-10 and DRSP scores and the overall degree of voice deviation.

Our analysis of the correlation between the investigated scales and the overall degree of voice deviation showed that the DRSP accurately represented the severity of dysphonia. These findings reinforce the applicability of this instrument for different laryngeal diagnoses.

## AUTHOR CONTRIBUTIONS

Nemr K participated in the design, structure of the research and supervision of the data collection, data analysis and manuscript writing. Cota A participated in the data collection, data analysis and manuscript writing. Tsuji D participated with the definition of the medical diagnoses of the sample. Simões-Zenari M participated in data analysis and manuscript writing.

## Figures and Tables

**Table 1:** Distribution of numerical data related to APA and AA.

Aspects analyzed	Mean	Median	Minimum	Maximum	SD	N
APA (0-100)						
Overall degree of voice deviation (D)	59.54	60.00	16	97	18.10	200
Roughness	54.74	52.50	6	95	17.26	200
Breathiness	44.57	42.00	0	94	19.46	200
Strain	25.39	25.00	0	94	25.89	200
Pitch	26.07	28.00	0	97	25.41	200
Loudness	19.61	0.00	0	96	24.42	200
Asthenia	1.17	0.00	0	60	7.45	200
Instability	4.58	0.00	0	96	16.45	200
Bitonal voice	1.12	0.00	0	75	7.79	200
Hypernasal voice	0.38	0.00	0	75	5.30	200
AA						
Jitter (%)	1.00	0.58	0.15	9.49	1.28	200
Shimmer (%)	4.74	3.33	0.92	20.54	4.08	200
Harmonics-to-noise ratio (dB)	19.90	20.79	2.84	32.00	6.5	200
Frequency of definition of harmonics (Hz)	1426.7	1327.0	0	4132.0	897.8	200

SD= Standard deviation.

**Table 2:** Distribution of categorical data related to APA and AA.

	Noise at low frequencies		Noise at high frequencies		Abnormalities in the harmonious series		Instability		Subharmonic		Frequency breaks		Voice breaks	
	N	%	N	%	N	%	N	%	N	%	N	%	N	%
Absent	57	28.5	7	3.5	8	4.0	119	59.5	114	57.0	166	83.0	147	73.5
Present	143	71.5	193	96.5	192	96.0	81	40.5	86	43.0	34	17.0	53	26.5
Total	200	100.0	200	100.0	200	100.0	200	100.0	200	100.0	200	100.0	200	100.0

**Table 3:** Correlation between D and the V-RQOL, VHI-10, and DRSP.

		Overall degree of voice deviation (D)
V-RQOL	Correlation coefficient	-0.206
	Significance (p)	0.003*
	N	200
VHI-10	Correlation coefficient	0.376
	Significance (p)	<0.001*
	N	200
DRSP	Correlation coefficient	0.177
	Significance (p)	0.012*
	N	200

Spearman’s correlation test; *statistically significant.

**Table 4:** Comparison between the DRSP-General score tertiles and the overall degree of voice deviation.

Overall degree of voice deviation (D)	DRSP score ranges			Kruskal-Wallis test (*p*-value)	Tukey’s multiple comparisons (*p*-value)
	1^st^ tertile (≤34.0)	2^nd^ tertile (≥34.1 and ≤52.0)	3^rd^ tertile (≥52.1)		
Mean	55.8	59.7	63.2		1^st^ X 2^nd^; *p*=0.425
Median	53.5	60.0	67.0	0.042*	1^st^ X 3^rd^; *p*=0.048*
SD	16.6	18.1	19.0		2^nd^ X 3^rd^; *p*=0.502
N	68	66	66		

* statistically significant; SD= Standard deviation.

**Table 5:** Correlation between the V-RQOL, VHI-10, and DRSP.

		V-RQOL	VHI-10
VHI-10	Correlation coefficient	-0.583	
	Significance (p)	<0.001*	
	N	200	
DRSP	Correlation coefficient	-0.396	0.626
	Significance (p)	<0.001*	<0.001*
	N	200	200

Spearman’s correlation test; *statistically significant.
